# A machine learning platform for the discovery of materials

**DOI:** 10.1186/s13321-021-00518-y

**Published:** 2021-05-27

**Authors:** Carl E. Belle, Vural Aksakalli, Salvy P. Russo

**Affiliations:** 1 ARC Centre of Excellence in Exciton Science, RMIT University, Melbourne 3000, Australia; 2 School of Science, RMIT University Australia, 124 La Trobe Street, 3000 Melbourne, Australia

**Keywords:** Machine learning, Deep learning, Materials prediction, Band gap

## Abstract

For photovoltaic materials, properties such as band gap $E_{g}$ are critical indicators of the material’s suitability to perform a desired function. Calculating $E_{g}$ is often performed using Density Functional Theory (DFT) methods, although more accurate calculation are performed using methods such as the GW approximation. DFT software often used to compute electronic properties includes applications such as VASP, CRYSTAL, CASTEP or Quantum Espresso. Depending on the unit cell size and symmetry of the material, these calculations can be computationally expensive. In this study, we present a new machine learning platform for the accurate prediction of properties such as $E_{g}$ of a wide range of materials.

## Introduction

Opportunities to harness the continued pace of computer processing capabilities as well as new and refined data processing techniques exist for those wishing to investigate and predict material properties computationally.

Using a Machine Learning (ML), Deep Learning (DL), and High Throughput (HT) computing techniques can provide an efficient robust data processing platform for the prediction and discovery of new materials.

ML techniques involve processing large datasets in order to generate highly accurate modelling algorithms that can be used to find relationships within the data and predict outcomes.

HT computing techniques involve aggregating the results of computations that have already been executed from many disparate data sources. Quantum chemical calculations and atomic scale calculations are often time consuming and CPU expensive, requiring hundreds of hours of super-computer processing time. Using pre-calculated results from these operations will greatly reduce processing time, allowing for a greater throughput on much more modest hardware.

The combination of ML with HT will allow for rapid and exhaustive exploration of materials properties within a computational environment, at a scale and speed that simply cannot be matched in a laboratory.

In this paper we present a bespoke software platform (codename: Hadoken) for the discovery of materials, as well as 5 models derived from ML techniques that can be used to accurately predict material properties (such as the band gap of a compound), and a newly developed website that provides the basis for a materials prediction platform.

## Deep learning

### Data preparation for deep learning

A dataset containing information about $250 \times 10^{3}$ simulations calculated via the Perdew-Burke-Ernzerhof (PBE [1, 2]) DFT functional using the projector augmented wave (PAW [3, 4]) method was sourced via the Hadoken platform and downloaded for processing.

#### Feature composition

The stoichiometry *S* value is a string which is split into its constituent parts (a form of one-hot encoding) and subsequently used to compose new features, comprising of the element and the count of the instance of that element. The one-hot encoding process involves decomposing categorical values into a binary representation.

1 $$\eqalign{& S({x_H},{x_{He}}, \ldots ,{x_{Og}}) \cr & \to \{ H = {x_H},He = {x_{He}}, \ldots ,Og = {x_{Og}}\} \cr}$$

To encode $$H_{2}O$$:

$$\begin{aligned}&S(H_{2}O) \Rightarrow S(H = 2, O = 1) \\&\rightarrow \{ H = 1, O = 2,\ldots , Og = 0 \} \end{aligned}$$

To encode copper indium selenide:

$$\begin{aligned}&S(CuInSe_{2}) \Rightarrow S(Cu_{1}In_{1}Se_{2}) \\&\rightarrow \{ H = 0,\ldots , Cu = 1, In = 1, Se = 2,\ldots , Og = 0 \} \end{aligned}$$

Isomers have the same stoichiometric *S* value, yet have differing physical structures ($C_{3}H_{4}$ for example). These isomers will produce identical encoding.

In this paper, the definition S (1) refers to this equation.

The gap type *GT* feature represents values that indicate the category (one-hot encoded) of gap type present in the compound.

2 $$\eqalign{ & GT({x_{G{T_{HM}}}},{x_{G{T_{ID}}}}, \ldots ,{x_{G{T_M}}}) \to \cr & \{ G{T_{HM}} = {x_{G{T_{ID}}}}, \ldots ,G{T_M} = {x_{G{T_M}}}\} x \in 0,1 \cr}$$

Table 1 details the possible gap type values with corresponding definitions.

**Table 1:** Possible gap type values with definitions

Value	Definition
NULL	No definition
HalfMetal	$^{*}$
InsulatorDirect	$^{*}$
InsulatorDirectSpinPolarised	$^{*}$
InsulatorIndirect	$^{*}$
InsulatorIndirectSpinPolarised	$^{*}$
Metal	$^{*}$

$^{*}$ Given a band gap, this keyword describes if the system is a metal, a semi-metal, an insulator with direct or indirect band gap [5].

The geometry *G* feature is decomposed using cell parameters (the unit cell’s lengths and angles) into 6 features:

3 $$\begin{aligned} G \rightarrow \{ a{\mathring{A}}, b{\mathring{A}}, c{\mathring{A}}, \alpha {^\circ }, \beta {^\circ }, \gamma {^\circ } \} \end{aligned}$$

Space group *SG* which defines one of the possible 230 symmetry groups of the crystal lattice is a categorical scalar that requires transformation into appropriate binary features (one-hot encoding). As an example, space group represents one of 230 possible categories with the use of a single integer: this scalar is transformed into 230 binary features:

4 $$\begin{aligned} \begin{aligned}SG(x_{SG_{1}}, x_{SG_{2}},\ldots , x_{SG_{230}}) \\\rightarrow \{ SG_{1} = x_{SG_{1}}, SG_{2} = x_{SG_{2}},\ldots , SG_{230} = x_{SG_{230}} \} \\x \in { 0, 1 } \end{aligned} \end{aligned}$$

To encode the space group 37:

$$\begin{aligned}&SG(37) \rightarrow \{ SG_{1} = 0,\ldots , SG_{37} = 1,\ldots , SG_{230} = 0 \} \end{aligned}$$

In the final stages of data preparation, constant features (features that contain the same value for each record) were dropped from the dataset, as well as any rows that contained null feature values. The dataset is now ready for use.

### Aggregated feature set

Data obtained from all databases (AFLOW [6], Materials Project [7]) is normalised and aggregated into a single, functional form. This process results in the aggregation of maximum number of homogeneous features from consumed data sources. Table 2 details the features obtained from the AFLOW and Materials Project databases along with example values.

**Table 2:** Feature set with names and example values

Name	Example
Stoichiometry	Al3Li3O12Si3
Band Gap	4.8022
Density	2.25761
Energy	− 151.631
Energy per Atom	− 7.22053
Fermi energy	0.4748
Geometry A, B, C	5.296, 5.296, 11.448
Geometry $\alpha$, $\beta$, $\gamma$	90, 90, 120
Space group	181

Table 3 details the attributes collected, along with names, example values, and original data source.

**Table 3:** Collated attribute set with example values and accompanying data source

Name	Example	AFLOW	Materials Project
Species	CaCuGeO	Species	Full_formula
Compound	Ca2Cu2Ge4O12	Compound	Full_formula ^a^
Band gap	1.2007	EGap	Band_gap
Density	4.60489	Density	Density
DFT type	1	dft_type^a^	If is_hubbard = true then PAW_PBE+U, else PAW_PBE
Energy	$-$121.07	Energy_cell	Energy
Energy per Atom	$-$6.05349	Energy_atom	Energy_per_atom
Fermi energy	3.4726	^b^	N/A
Gap type	InsulatorIndirect	Egap_type	N/A
Geometry A	6.949605	Geometry ^a^	N/A
Geometry B	6.949605	Geometry ^a^	N/A
Geometry C	5.44499	Geometry ^a^	N/A
Geometry alpha	76.82593	Geometry ^a^	N/A
Geometry beta	76.82593	Geometry ^a^	N/A
Geometry gamma	83.10932	Geometry ^a^	N/A
K-Space	$\Gamma$-Y-F-L-Z-...-N-Z-$F_{1}$	kpoints ^a^	N/A
Number of atoms	20	natoms	nsites
Space group	15	Spacegroup_orig	Spacegroup
Volume	248.674	Volume_cell	Volume

^a^ Post processing applied

^b^Sourced from associated files

Table 2 details the feature set with names and example values.

### Additional feature set derivation

Additional features useful for ML can be derived from existing features and also user input. Deriving these features frees the user from the necessity of performing these calculations and expedites work flow. In some instances, derivation of these additional features has been undertaken purely for experimental purposes, with the expectation that further refinement in the future will yield less theoretical results.

In this paper, the notation S (1) refers to *S* provided by the Definition 1.

#### Number of atoms

The total number of atoms *N* contained within the system can be derived from S (1) such:

5 $$\begin{aligned} N = \sum S(x_{i}) \end{aligned}$$

where S (1) describes the stoichiometric composition of the material. This feature returns the sum of species of each atom contained in the unit cell multiplied by the instance.

#### Atomic weight

The total atomic weight $T_{Ar}$ of the system with reference to S (1) is given by:

6 $$\begin{aligned} T_{Ar} = \sum Ar_{i} \times S(x_{i}) \end{aligned}$$

where S (1) describes the stoichiometric composition of the material and *Ar* [8] describes the atomic weight of each element. This feature returns the sum of each atomic weight of each species considered individually in the unit cell multiplied by the instance.

#### Chemical potential

The total chemical potential $T_{\mu }$ of the system with reference to S (1) is given by:

7 $$\begin{aligned} T_{\mu } = \sum \mu _{i} \times S(x_{i}) \end{aligned}$$

where S(1) describes the stoichiometric composition of the material and $\mu$ describes the chemical potential of each element. This feature returns the sum of each chemical potential of each species considered individually in the unit cell multiplied by the instance. This feature contains values generated by the software given a stoichiometry value. The chemical potential values are provided from the corresponding VASP POTCAR files.

#### S, P, D, F electrons

The total count of the number of electrons $T_{e}$ in each type of sub shell within the compound is given by:

8 $$\begin{aligned} T_{e} = \sum e_{i} \end{aligned}$$

where $e_{i}$ describes the number of electrons present in the corresponding sub shell. Electron configuration is determined using values from the literature [9].

#### S, P, D, F orbitals

The total count of each type of orbital $T_{\sigma }$ within the compound is given by:

9 $$\begin{aligned} T_{\sigma } = \sum \sigma _{i} \end{aligned}$$

where $\sigma _{i}$ describes the corresponding number of orbitals present in the element. Orbital configuration is determined using values from the literature [9].

#### Symmetry

The symmetry elements *HS* [10] associated with the space group of the crystal lattice has been stored in our database. This information is one-hot encoded in a similar fashion to SG (4).

10 $$\begin{aligned} \begin{aligned}HS(x_{HS_{1}}, x_{HS_{2}},\ldots , x_{HS_{63M}}) \\\rightarrow \{ HS_{1} = x_{HS_{1}}, HS_{2} = x_{HS_{2}},\ldots , HS_{63M} = x_{HS_{63M}} \} \\x \in { 0, 1 } \end{aligned} \end{aligned}$$

#### Electron affinity

The total electron affinity $T_{EA}$ with reference to S (1) is given by:

11 $$\begin{aligned} T_{EA} = \sum EA_{i} \times S(x_{i}) \end{aligned}$$

where S (1) describes the stoichiometric composition of the material and $EA_{i}$ describes the electron affinity [11] of each element. This feature returns the sum of each electron affinity of each species considered individually in the unit cell multiplied by the instance.

#### Electronegativity

The total electronegativity $\chi$ is given by the Mulliken electronegativity definition [12, 13]:

12 $$\begin{aligned} \chi = \sum \frac{E_{i} + E_{ea}}{2} \end{aligned}$$

where $E_{i}$ describes the first ionisation energy [14] and $E_{ea}$ describes the electron affinity [11].

#### Ionisation energy

The total ionisation energy $T_{IE}$ with reference to S (1) is given by:

13 $$\begin{aligned} T_{IE} = \sum IE_{i} \times S(x_{i}) \end{aligned}$$

where S (1) describes the stoichiometric composition of the material and $IE_{i}$ describes the ionisation energy [14] of each element.

#### Mass density

The total mass density $T_{\rho }$ with reference to S (1) is given by:

14 $$\begin{aligned} T_{\rho } = \sum \rho _{i} \times S(x_{i}) \end{aligned}$$

where S (1) describes the stoichiometric composition of the material and $\rho _{i}$ describes the density [15, 9] of each element. This feature returns the sum of each mass density value multiplied by the instance count of the corresponding element.

#### Valence electrons

The total number of valence electrons $T_{Ve}$ with reference to S (1) is given by:

15 $$\begin{aligned} T_{Ve} = \sum Ve_{i} \times S(x_{i}) \end{aligned}$$

where S (1) describes the stoichiometric composition of the material and $Ve_{i}$ describes the number of valences electrons present for each element [15]. Currently, the number of valence electrons is determined primarily from the specification of the chemical elements in the VASP POTCAR file associated with the structure.

#### Effective mass

For a free electron, effective mass [16, 17] is given by

16 $$\begin{aligned} E = \frac{\hbar ^{2} k^{2}}{2m_{e}} \end{aligned}$$

For an electron in a crystal, the effective mass approximation is given by

17 $$\begin{aligned} E' = \frac{\hbar ^{2} k^{2}}{2m'_{e}} \end{aligned}$$

where $m'_{e} = xm_{e}$. Thus the dispersion may be rewritten as

18 $$\begin{aligned} E' = \frac{1^{2} \dot{k}^{2}}{2(x \dot{1})} = \frac{k^{2}}{2x} \end{aligned}$$

Using the second derivative of (18) to calculate *x*

19 $$\begin{aligned} \frac{d^{2}E'}{dk^{2}} = \frac{d}{dk}\left( \frac{dE'}{dk}\right) = \frac{d}{dk}\left( \frac{k}{x}\right) = \frac{1}{x} \end{aligned}$$

Fitting a curve to the conduction band minima of an *E*-*k* diagram using the form $y = ax^{2} + bx + c$ yields

20 $$\begin{aligned} E' = ak^{2} + bk + c \end{aligned}$$

Then

21 $$\begin{aligned} \frac{d^{2}E'}{dk^{2}} = 2a \end{aligned}$$

And

22 $$\begin{aligned} x = \left( \frac{d^{2}E'}{dk^{2}}\right) ^{-1} = (2a)^{-1} \end{aligned}$$

Thus our final equation for calculating effective mass (adjusting for atomic units) is given by:

23 $$\begin{aligned} m^{*} = (2a)^{-1} \end{aligned}$$

The VASP software package can produce EIGENVAL files which contain the Kohn-Sham eigenvalues for all *k*-points. We have developed software to parse these files and produce the appropriate band structure diagrams, to which a parabola may be fitted. The EIGENVALS output usually appears in the following format:

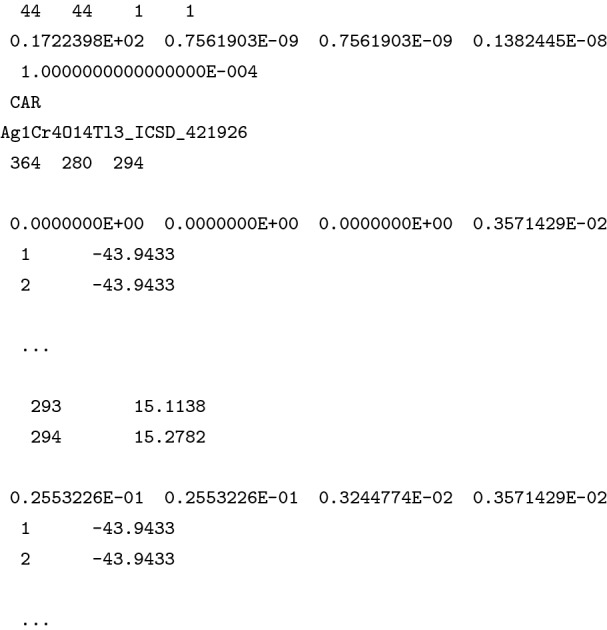



The following line in this file contains important information required during processing:

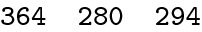



The values on this line are the number of electrons, number of *k*-points, and number of bands respectively. Lines that contain 4 double-values contain information regarding the 3-dimensional position in *k*-space (*x*, *y*, *z*), as well as a weighting factor (not used by our software):

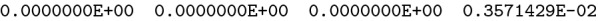



These values are parsed into a vector and stored in memory. Immediately following the coordinate lines are lines containing energies associated at that coordinate:

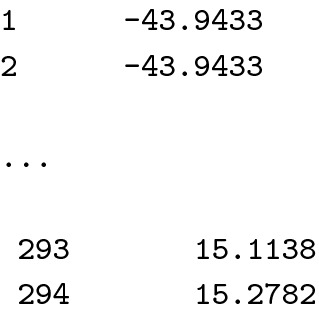



Coordinate vectors represent direct coordinate values (*x*, *y*, *z*) and are require further processing to be useful for $m^{*}$ calculation.

24 $$\begin{aligned} \begin{aligned}f(\alpha ) = (2 \times \pi \times \alpha ) \\\quad .^.. \\x' = f(x) \\y' = f(y) \\z' = f(z) \\ \end{aligned} \end{aligned}$$

The reciprocal lattice is a $3 \times 3$ matrix defined as

25 $$\begin{aligned} G_{m} = m_{1}b_{1} + m_{2}b_{2} + m_{3}b_{3} \end{aligned}$$

where the reciprocal primitive vectors are defined as

26 $$\begin{aligned} \begin{aligned} b_{1}&= \alpha {{\hat{i}}}_{1} + \beta {{\hat{j}}}_{1} + \gamma {{\hat{k}}}_{1} \\ b_{2}&= \alpha {{\hat{i}}}_{2} + \beta {{\hat{j}}}_{2} + \gamma {{\hat{k}}}_{2} \\ b_{3}&= \alpha {{\hat{i}}}_{3} + \beta {{\hat{j}}}_{3} + \gamma {{\hat{k}}}_{3} \end{aligned} \end{aligned}$$

The reciprocal lattice (values sourced from the OUTCAR file, another VASP output file) is used to transform coordinate vectors $\mathbf {v}_{i} = [x'_{i} y'_{i} z'_{i}]$ by the reciprocal lattice such:

27 $$\begin{aligned} \mathbf {v}_{i} = \begin{bmatrix} x'_{i}&y'_{i}&z'_{i} \end{bmatrix}^{T} \times G_{m} \end{aligned}$$

Finally, the distance between two 3-dimensional *k*-coordinate vectors $\mathbf {v}_{i}$ and $\mathbf {v}_{j}$ is described by:

28 $$\begin{aligned} d = \sqrt{(\mathbf {v}_{jx} - \mathbf {v}_{ix})^{2} + (\mathbf {v}_{jy} - \mathbf {v}_{iy})^{2} + (\mathbf {v}_{jz} - \mathbf {v}_{iz})^{2}} \end{aligned}$$

This value is used as the *k*-value (converted from units of ${\AA }^{-1} \rightarrow {\mu _{B}}^{-1}$) along the *x*-axis in the following *E*-*k* diagram, with *E* (converted from units of $eV \rightarrow Ha$) comprising the *y*-axis values. This process ostensibly provides energy values that correspond to the associated position in the Brillioun zone.

**Fig. 1:**
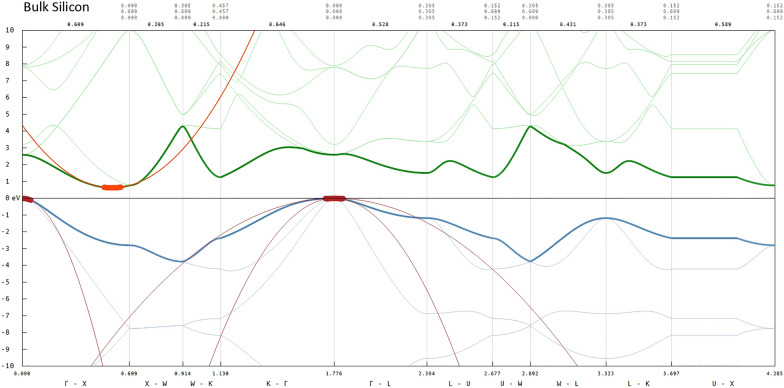
Bulk silicon band structure

Figure 1 shows the band structure (*E*-*k*) diagram of $Si_{2}$ generated by the Hadoken software. Conduction bands are shaded green, with the lowest unoccupied molecular orbital (LUMO) is shown as bold green. Valence bands are shaded blue, with the highest occupied molecular orbital (HOMO) show as bold blue. An orange parabola has been fitted to the LUMO minimum in the $\Gamma$-*X* segment, and it is this curve that is used to calculate effective mass. Also shown are red parabolae fitted to the HOMO maxima.

Fitting a parabola in the quadratic form $y = ax^{2} + bx + c$ yields the coefficient *a* which can then be used by (23) to obtained the final $m^{*}$ value.

Should more than one fit per *k*-space segment be possible, then the resultant values are averaged to yield the final effective mass value. Currently, only $m^{*}$ values calculated in the $\Gamma$-*X* segment via this method are persisted.

The following Table 4 displays the entire feature set, including sourced and derived values, corresponding example values and units.

**Table 4:** Full feature set with example values and accompanying units

Name	Example	Units	Data type	Aggregated	Calculated
Species	CaCuGeO		String	Yes	No
Compound	Ca2Cu2Ge4O12		String	Yes	No
Band gap	1.2007	eV	Double	Yes	No
Density	4.60489	eV	Double	Yes	No
DFT type	1		Int32	Yes	No
Energy	− 121.07	eV	Double	Yes	No
Energy per Atom	− 6.05349	eV	Double	Yes	No
Fermi energy	3.4726	eV	Double	Yes	No
Gap type	InsulatorIndirect		String	Yes	No
Geometry A	6.949605	Å	Double	Yes	No
Geometry B	6.949605	Å	Double	Yes	No
Geometry C	5.44499	Å	Double	Yes	No
Geometry alpha	76.82593	Degrees	Double	Yes	No
Geometry beta	76.82593	Degrees	Double	Yes	No
Geometry gamma	83.10932	Degrees	Double	Yes	No
K$-$Space	$\Gamma$-Y-F-L-Z-...-N-Z-$F_{1}$		String	Yes	No
Number of atoms	20		Int32	Yes	No
Space group	15		Int32	Yes	No
Volume	248.674	Å$^{3}$ or $\hbox {Bohr}^{3}$ [18]	Double	Yes	No
Effective mass	0 or NULL		Double	No	Yes
Total atomic weight	689.756		Double	No	Yes
Total chemical potential	− 8390.4896		Double	No	Yes
Total electron affinity	24.9832632		Double	No	Yes
Total electro negativity	6.19191658	kJ/mol	Double	No	Yes
Total ionisation energy	222.6934	eV	Double	No	Yes
Total density	42.309148	eV	Double	No	Yes
Total number of S Orbitals	56		Int32	No	Yes
Total number of P Orbitals	96		Int32	No	Yes
Total number of D Orbitals	30		Int32	No	Yes
Total number of F Orbitals	0		Int32	No	Yes
Total number of electrons	322		Int32	No	Yes
Total number of S electrons	110		Int32	No	Yes
Total number of P electrons	152		Int32	No	Yes
Total number of D electrons	60		Int32	No	Yes
Total number of F electrons	0		Int32	No	Yes
Valence electrons	94		Int32	No	Yes

### Deep learning model training process

All models were trained using the same process:

1. Features in the entire 0.477 GB dataset were normalised.
2. Data was split into two streams: training and validation at a ratio of 0.7/0.3.
3. An artificial neural network based on a sequential DL model from the Keras framework on a TensorFlow back end with dense layers (100, 50) was used and trained over 300 iterations.
4. Verification that over-fitting was not occurring. Over-fitting is characterised by an increase in loss which will be reflected in the training history. Even after 300 iterations, the loss recorded continues to converge, indicating that the algorithm is still learning (i.e., not over-fitting). Had the training process resulted in an increase in loss, we could be sure over-fitting was occurring.
5. The neural network was optimised by training it with all the data over 1000 iterations. The following Table 5 demonstrates that the optimisation process may yield extra accuracy when training a model for production deployment.
6. Information about the neural network was serialised for production use (layers, weights, biases, activation functions etc.).
7. Optimised models are persisted for future use via the https://www.hadokenmaterials.io/ website and associated API

The models described in this document have been made available for use at https://www.hadokenmaterials.io/ with the API documentation available at https://www.hadokenmaterials.io/Home/Api. These models are also made available via GitHub at https://github.com/carlyman77/MaterialsDiscoveryML.

**Table 5:** Comparison of unoptimised ML and optimised Models

Model	State	MAE	RMSE	$\hbox {R}^{2}$	99%
Band Gap-single	Unoptimised	0.079572	0.297179	0.914471	3.044744
Band Gap-single	Optimised	0.057742	0.214150	0.955388	2.315024
Band Gap-minimal	Unoptimised	0.072204	0.300485	0.912558	3.212371
Band Gap-minimal	Optimised	0.045086	0.162154	0.974421	1.590898
Band Gap-maximal	Unoptimised	0.082444	0.311686	0.905917	3.232445
Band Gap-maximal	Optimised	0.046388	0.175463	0.970050	1.946711
Fermi energy	Unoptimised	0.249163	0.379878	0.975808	2.765868
Fermi energy	Optimised	0.308781	0.392329	0.974224	2.141287

### Determination of model accuracy

We include three different loss functions used to determine accuracy for the predictive models, and a single loss function for the classification model. All metrics should be considered when evaluating the accuracy of a model, as each method has advantages in certain applications. For example, if the average errors are evenly distributed then both Mean Absolute Error and Root Mean Squared Error outputs should converge. However, Root Mean Squared Error will penalise large outlier errors as the errors are squared before an average is taken.

#### Mean absolute error (MAE)

This value is derived from the mean_absolute_error [19] function which produces a risk metric corresponding to the expected value of the absolute error loss or *l*1-norm loss.

Given ${\hat{y}}_{i}$ to be the predicted value of the *i*-th sample, and $y_{i}$ to be the corresponding true value, then the MAE estimated over *n* samples, is defined such that:

29 $$\begin{aligned} MAE(y, {\hat{y}}) = \frac{1}{n} \sum ^{n - 1}_{i = 0} |y_{i} - {\hat{y}}_i| \end{aligned}$$

#### Root mean squared error (RMSE)

This value is derived by taking the square root of the Mean Squared Error (MSE, quadratic or L2 loss) value generated by the mean_squared_error [20] function.

Given ${\hat{y}}_{i}$ to be the predicted value of the *i*-th sample, and $y_{i}$ to be the corresponding true value, then the MSE estimated over *n* samples, is defined such:

30 $$\begin{aligned} MSE(y, {\hat{y}}) = \frac{1}{n} \sum _{i = 0}^{n - 1}(y_{i} - {\hat{y}}_{i})^{2} \end{aligned}$$

Therefore:

31 $$\begin{aligned} RMSE(y, {\hat{y}}) = \sqrt{\frac{1}{n} \sum _{i = 0}^{n - 1}(y_{i} - {\hat{y}}_{i})^{2}} \end{aligned}$$

#### $\hbox {R}^{2}$

This value is derived from the r2_score [21] function which is a representation of the proportion of explained variance. A perfect score is 1.0 which indicates that all independent variables are used to explain variation in the dependant variable.

Given ${\hat{y}}_{i}$ to be the predicted value of the *i*-th sample, and $y_{i}$ to be the corresponding true value for a total of *n* samples, then the estimated $R^{2}$ is defined such:

32 $$\begin{aligned} R^{2}(y, {\hat{y}}) = 1 - \frac{\sum ^{n}_{i = 1}(y_{i} - {\hat{y}}_{i})^{2}}{\sum ^{n}_{i = 1}(y_{i} - {\bar{y}})^{2})} \end{aligned}$$

where $${\bar{y}} = \frac{1}{n} \sum ^{n}_{i = 1} y_{i}$$ and $$\sum ^n_{i = 1}(y_{i} - {\hat{y}}_i)^{2} = \sum ^n_{i = 1} \epsilon ^{2}_{i}.$$

## Modelling and results

### Overview

Models are produced by the ML training process, and contain the refined weights, biases and activation functions required to operate independently of the original dataset. Models are software assets that can be used to perform complex algorithmic tasks such as prediction or classification.

### Band gap

Band gap $E_{g}$ is an energy range between the uppermost valence band (valence band maximum) and the lowest conduction band (conduction band minimum) of a crystal. Electrons in the valence bands can transition into the conduction bands upon excitation. This size of the band gap is a critical feature that many of the material’s possible applications.

Photovoltaic (PV) materials are semiconductors, and so it follows that $E_{g}$ is a key metric when considering a material’s suitability for PV applications.

#### Deep learning to predict band gap (Single feature)

This model attempts to predict $E_{g}$ from stoichiometry only. This model uses a single feature, stoichiometry S (1), such:

$$\begin{aligned} E_{g}(S) = M(S) \end{aligned}$$

where $E_{g}(S)$ describes the predicted result computed by *M* from S (1).

#### Results

**Fig. 2:**
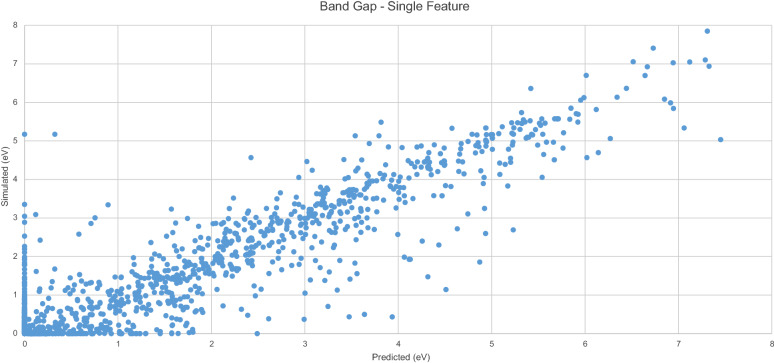
Simulated vs. predicted $E_{g}$ -single feature

Figure 2 displays the predicted $E_{g}$

values generated by the model with the original $E_{g}$ values. A clear linear trend is evident.

**Fig. 3:**
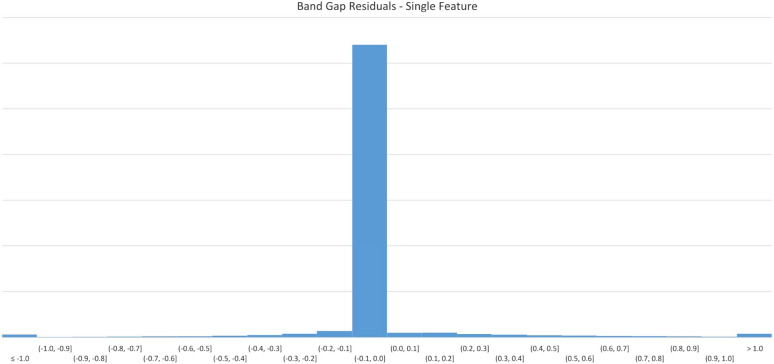
$E_{g}$ Model residuals-single feature

Figure 3 displays errors in 0.1 eV buckets. The majority of predicted results appear in the first negative bucket, indicating that for most predictions, the resultant value is no more than 0.1 eV different from the original value.

**Fig. 4:**
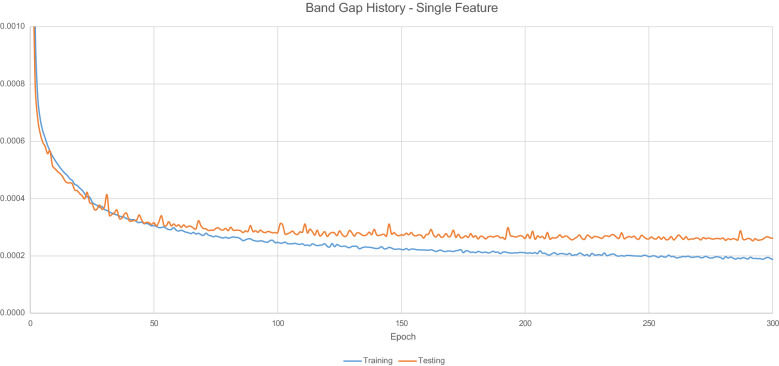
$E_{g}$ Model Training History - Single Feature

Figure 4 displays the loss values generated by during the model training process.

Table 6 details the overall predictive accuracy metrics for the model.

**Table 6:** Single feature model performance metrics

Name	Value
Mean absolute error	0.079572
Root mean squared error	0.297179
$\hbox {R}^{2}$	0.914471
99% Quantile error	3.044744

#### Deep learning to predict band gap (minimal features)

This model attempts to predict $E_{g}$ from the fewest features considered logical that are also easily sourced, i.e., they can be found in literature and/or do not require complex computation to derive. This model uses the feature geometry *G* which is decomposed into cell parameters (the unit cell’s lengths and angles).

This model uses 3 main features, stoichiometry S (1), geometry G (3), and space group SG (4), such:

$$\begin{aligned} E_{g}(S, G, SG) = M(S, G, SG) \end{aligned}$$

where $E_{g}(S)$ describes the predicted result computed by *M* from S (1). G (3), and SG (4).

#### Results

**Fig. 5:**
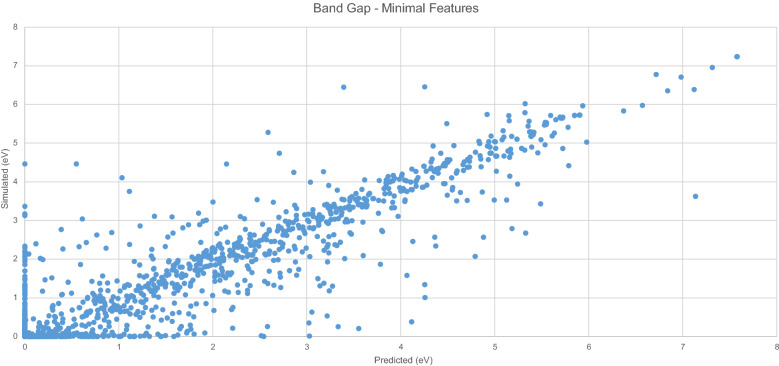
Simulated vs. predicted $E_{g}$ - minimal features

Figure 5 displays the predicted $E_{g}$ values generated by the model with the original $E_{g}$ values. A clear linear trend is evident, and the spread of data points from this trend is much less than the previous model.

**Fig. 6:**
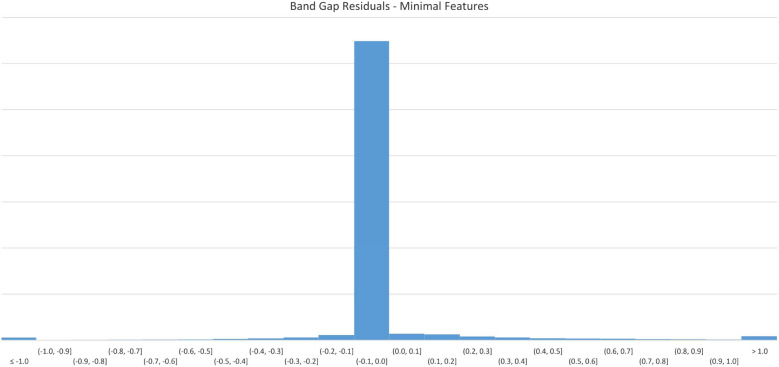
$E_{g}$ Model residuals-minimal features

Figure 6 displays errors in 0.1 eV buckets. The majority of predicted results appear in the first negative bucket, indicating that for most predictions, the resultant value is no more than 0.1 eV different from the original value.

**Fig. 7:**
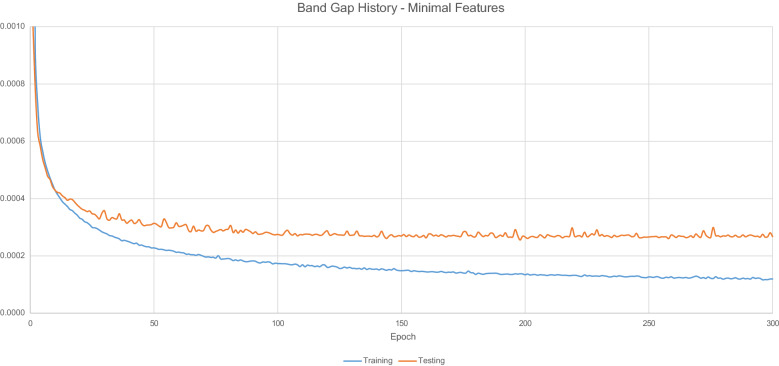
$E_{g}$ Model training history-minimal features

Figure 7 displays the loss values generated by during the model training process.

Table 7 details the overall predictive accuracy metrics for the model.

**Table 7:** Minimal feature model performance metrics

Name	Value
Mean absolute error	0.072204
Root mean squared error	0.300485
$\hbox {R}^{2}$	0.912558
99% Quantile error	3.212371

#### Deep learning to predict band gap (maximal features)

This model attempts to predict $E_{g}$ from the maximum number of features available from the collated dataset. This model is described as such:

$$\begin{aligned} E_{g}(F_{ALL}) = M(F_{ALL}) \end{aligned}$$

where $E_{g}(F_{ALL})$ describes the predicted result computed by *M* from $F_{ALL}$, and $F_{ALL}$ describes all features in the dataset.

#### Results

**Fig. 8:**
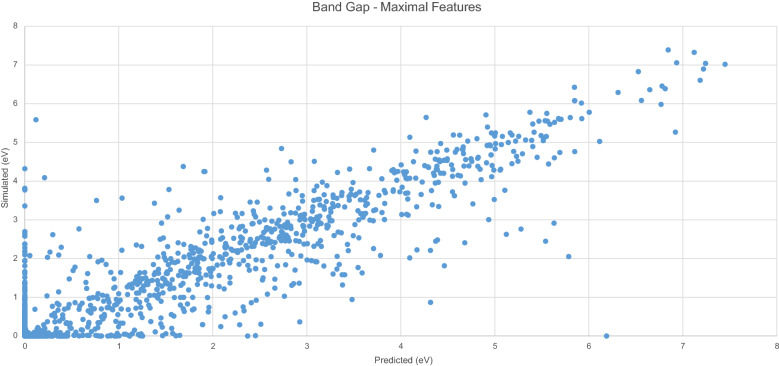
Simulated vs. predicted $E_{g}$ -maximal features

Figure 8 displays the predicted $E_{g}$ values generated by the model with the original $E_{g}$ values. A clear linear trend is evident, with the spread of data points from this trend much similar to the previous model.

**Fig. 9:**
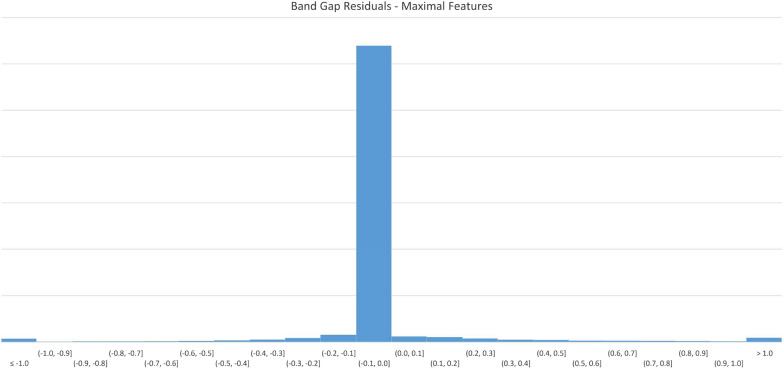
$E_{g}$ Model residuals-maximal features

Figure 9 displays errors in 0.1 eV buckets. As with the previous model, the majority of predicted results appear in the first negative bucket, indicating that for most predictions, the resultant value is no more than 0.1 eV different from the original value.

**Fig. 10:**
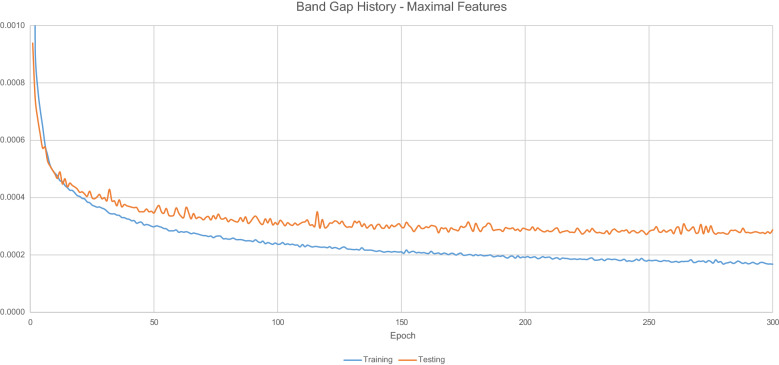
$E_{g}$ Model training history-maximal features

Figure 10 displays the loss values generated by during the model training process.

Table 8 details the overall predictive accuracy metrics for the model.

**Table 8:** Maximal feature model performance metrics

Name	Value
Mean absolute error	0.082444
Root mean squared error	0.311686
$\hbox {R}^{2}$	0.905917
99% Quantile error	3.232445

#### Comparison among deep learning models

Table 9 summarises the predictive accuracy metrics for each model. All 3 models are extremely accurate, and of note is the diminishing returns realised by the addition of many extra features: the model using a single feature is almost as accurate as the model that uses 20 features.

**Table 9:** Comparison metrics with feature and encoded feature count

Name	F	$\hbox {F}_{{E}}$	MAE	RMSE	$\hbox {R}^{2}$	99%
Single	1	100	0.079572	0.297179	0.914471	3.044744
Minimal	8	311	0.072204	0.300485	0.912558	3.212371
Maximal	20	348	0.082444	0.311686	0.905917	3.232445

### Fermi energy

Fermi energy is also an attribute useful for the design and discovery of materials, however some online data sources do not store this value. We provide a model for the prediction of this property.

#### Deep learning to predict fermi energy

This model attempts to predict $E_{F}$ from the fewest features. This model uses 2 main features, stoichiometry (one-hot encoded) S (1), and geometry G (3). This model is described as:

$$\begin{aligned} E_{F}(S, G) = M(S, G) \end{aligned}$$

#### Results

**Fig. 11:**
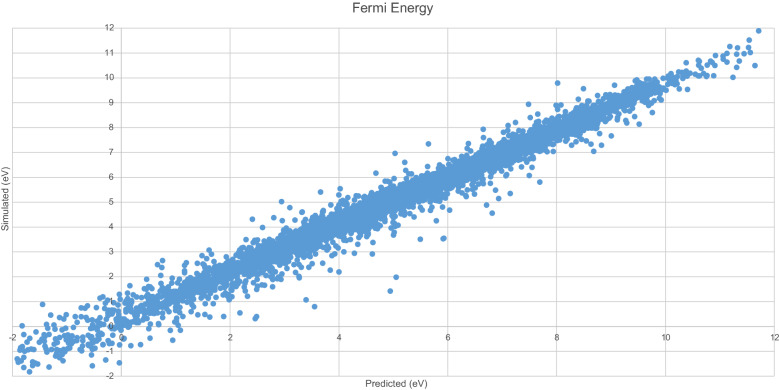
Simulated vs. predicted $E_{F}$

Figure 11 displays the predicted $E_{F}$ values generated by the model with the original $E_{F}$ values. A clear linear trend is evident, with most data points clustered on or around this trend.

**Fig. 12:**
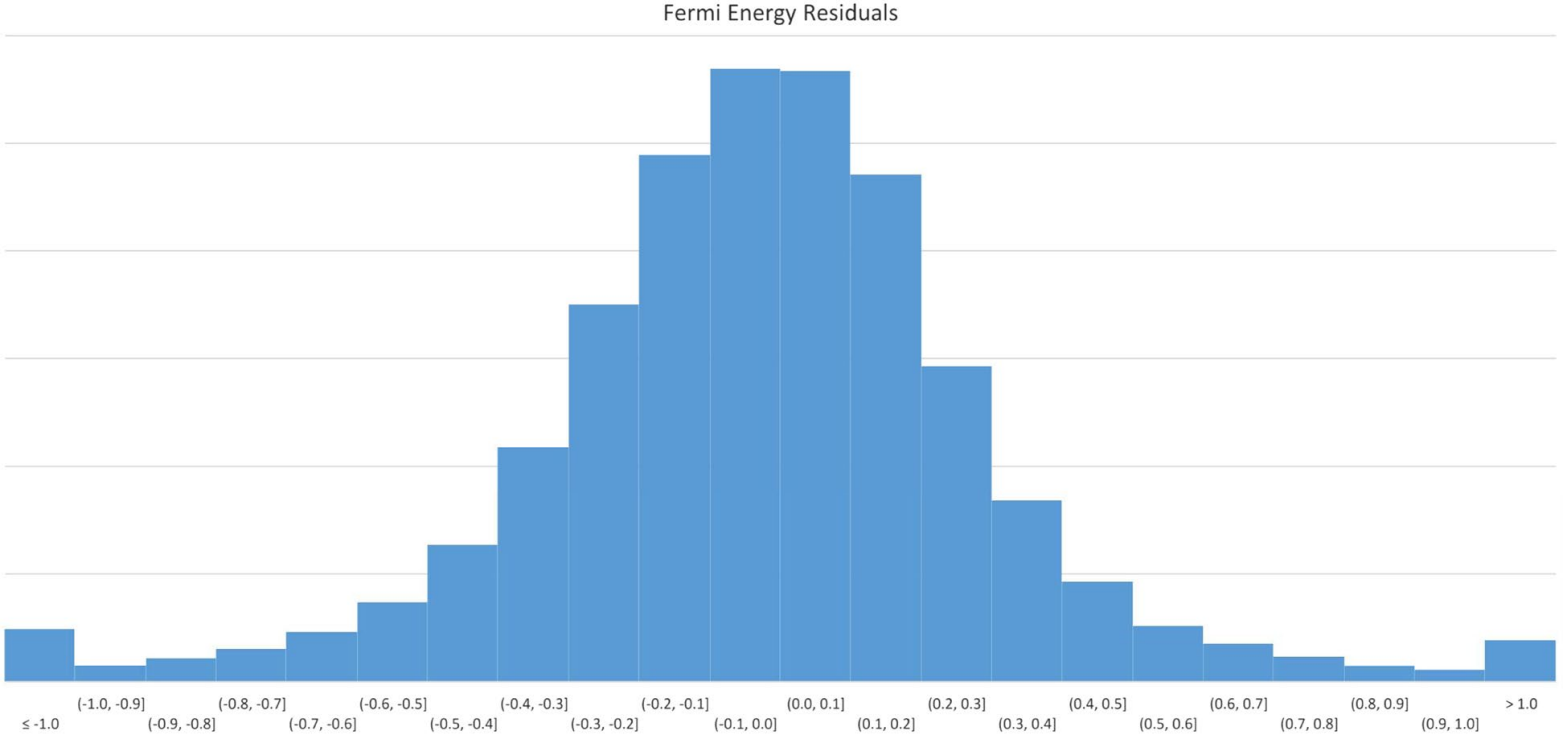
$$E_{F}$$ Model residuals

Figure 12 displays errors in 0.1 eV buckets. This model is accurate to within 0.5 eV for the majority of predicted values.

**Fig. 13:**
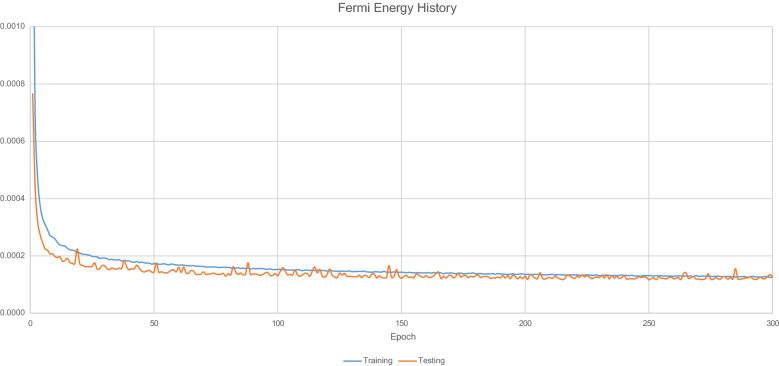
$E_{F}$ Model training hstory

Figure 13 displays the loss values generated by during the model training process.

Table 10 details the overall predictive accuracy metrics for the model.

**Table 10:** Fermi energy model performance metrics

Name	Value
Mean absolute error	0.249163
Root mean squared error	0.379878
$\hbox {R}^{2}$	0.975808
99% quantile error	2.765868

### Gap type

Gap type is an important attribute used to classify the type of band gap present in a material. Typically the gap type relates directly to the usefulness of a material for a specific application. For example, metals have no band gap and and such make excellent conductors, whilst semiconductors may have a direct or indirect band gap (an indirect band gap is characterised by the phonon-assisted transmission). Insulators typically have a very large band gap.

#### Deep learning to classify gap type

This model uses 2 main features, stoichiometry (one-hot encoded) S (1), and space group SG (4), that are encoded (or decomposed) into values of varying size. This model is described as:

$$\begin{aligned} GapType(S, SG) = M(S, SG) \end{aligned}$$

#### Results

**Fig. 14:**
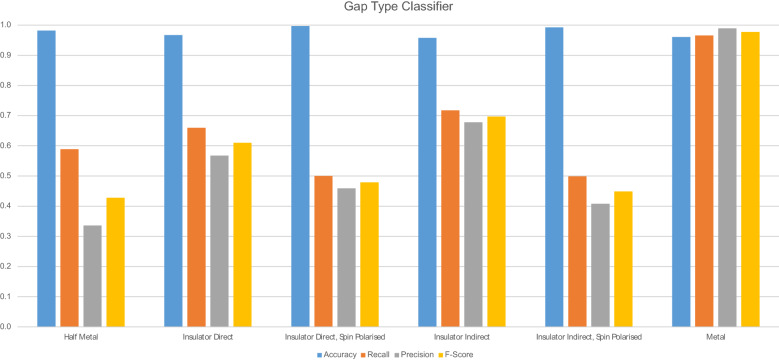
Gap type class metrics

Figure 14 displays the accuracy of gap type predictions per gap type. This model is most useful at predicting whether a gap type is an direct insulator or a metal.

**Fig. 15:**
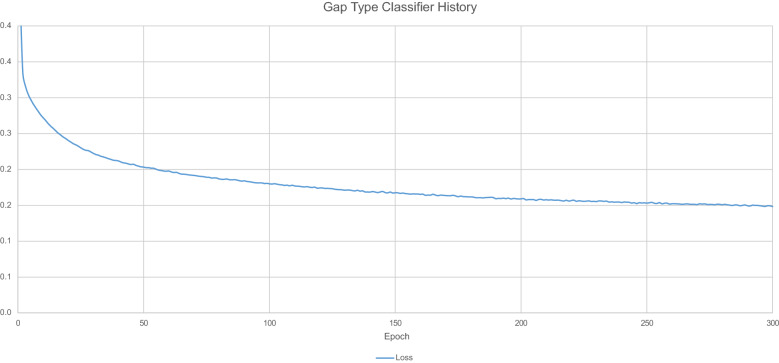
Gap type classification training history

Figure 15 displays the loss values generated by during the model training process.

Table 11 details the overall predictive accuracy metrics for the model.

**Table 11:** Gap type classifier model performance metrics

Name	Value
$\hbox {R}^{2}$	0.924429

Table 12 details the metrics for each class of the model.

**Table 12:** Gap type classifier model performance metrics per class

Class	Accuracy	Recall	Precision	F-Score
Half metal	0.981922	0.588644	0.336201	0.427970
Insulator direct	0.967027	0.659524	0.567789	0.610228
Insulator direct, Spin Polarised	0.9967527	0.500000	0.459016	0.478632
Insulator indirect	0.957695	0.717451	0.678095	0.697218
Insulator Indirect, Spin Polarised	0.992452	0.498920	0.408127	0.448980
Metal	0.960757	0.965741	0.989014	0.977239

## Production deployment of machine learning models

In addition to development of the preceding models, we have developed a lightweight and efficient method for deploying models to a production environment.

Multiple files are produced by Keras when persisting a model, namely the weights and structure of the network. The weights are stored in the HDF5 [22] format and the model structure in a JSON format, neither of which are suitable for a number of reasons: JSON offers no schema support, or mature query language, comments, or meta-data. JSON is also a terse format designed to be used when the contract is pre-agreed upon, and therefore does not make a good candidate to support rich, searchable data models. The HDF5 format is not human readable and is not easily parsed. Unifying these two files in a more appropriate format is a welcome improvement.

In addition to this, no information is saved with the model about how it is intended to be used. For example, inputs are not labelled, and no normalising parameters are included, which renders the model not portable and useless for production consumption. To address this, we have developed a simple, portable XML format that is searchable and can be validated against a schema. Only a single file is required to instantiate a usable model in a production environment that is guaranteed to produce reliable results from minimal code.

### Artificial neural network function

Provisioning of ML models from the XML definition is provided via the Hadoken.ML.NeuralNetwork type located in the Hadoken.ML assembly. This custom-built Artificial Neural Network (ANN) functions as a series of completely connected layers using the following method:

1. Inputs are multiplied by weights and forwarded to the nodes in each layer
2. Each node introduces a bias and another weight and sends the value to the next layer via the activation function

**Fig. 16:**
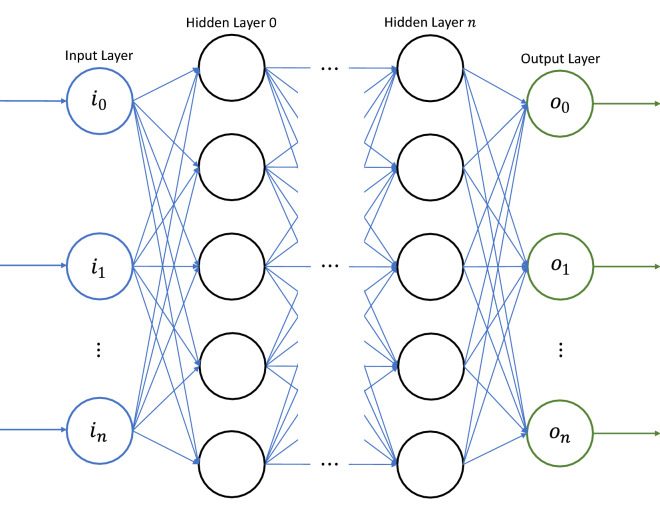
Neural network function

Figure 16 displays the map of a typical neural network. Inputs are fully connected with the first hidden layer, which is in turn fully connected to each following layer. This process is completed for each hidden layer, with results forwarded to the output layer.

**Fig. 17:**
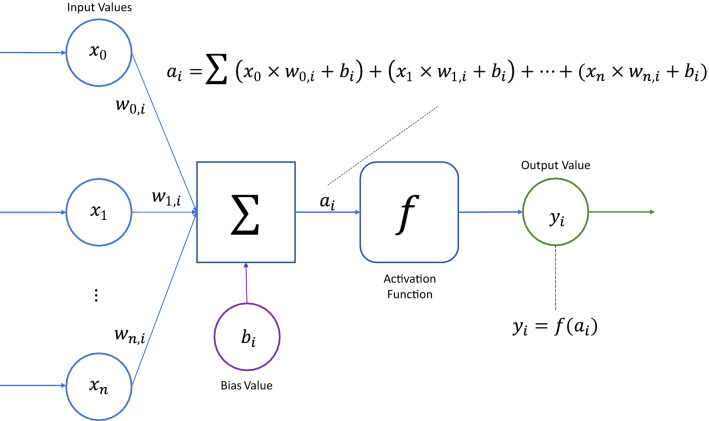
Neural network node function

Figure 17 displays the map of a neural network node. Inputs are multiplied by a weight and then added to a bias value. The sum of these operations is forwarded to an activation function which determines the final output value.

### Supported activation functions

Table 13 details the activation functions provided by the software.

**Table 13:** Supported activation functions

Name	Form
Hyperbolic tangent	$f(x) = \tanh x$
Rectified linear unit	$f(x) = \max \{ 0, x \}$
Sigmoid	$f(x) = \frac{1}{1 + e^{-1}}$
Softmax	$f(x_{i}) = \frac{e^{x_{i}}}{\sum _{j}e^{x_{j}}}$

### Supported normalisers

Table 14 details the normalisers provided by the software.

**Table 14:** Supported normalisers

Name	Form
Mean	$y = \frac{x - {{\,\mathrm{mean}\,}}{x}}{\max {x} - \min {x}}$
Min/Max	$y = \frac{x - \min {x}}{\max {x} - \min {x}}$

## Software platform

### Architecture

A bespoke software platform (codename: Hadoken) was created for the express purpose of aggregating materials data from disparate representational state transfer (REST) APIs such as Materials Project [7] and AFLOW [6]. Data from these sources is collected via an aggregator and stored in a relational database. Additional supporting files that may be of use (such as associated VASP [23] files) are also downloaded and stored for later use. Useful attributes such as Fermi energy $E_{F}$ that are not present in REST API data are sourced from the VASP files and added to the database. Curated data is then used for the purposes of training ML models for predictive tasks.

### Technology stack

The technology stack mirrors current popular industry standard for rapid application development (RAD), and is based on Microsoft’s .NET Core Framework [24] and Microsoft SQL Server 2017 [25]. ML technologies include Python 3.5 [26] and TensorFlow [27] as well as Azure ML Studio [28].

### Data collection

Data are initially sourced from two streams, on-line and off-line. On-line data sources are actively maintained network resources which release edits in real (or near to real) time. These often take the form of REST web API offerings including Materials Project and AFLOW. Some of these web services include information gathered from other sources, such as the Inorganic Crystal Structure Database (ICSD) [29].

These RESTful web services provide an industry standard method for querying and retrieving data. Data is provided in JSON format, which is then parsed into a common object model and stored locally. On-line data sources are much easier to work with than off-line, as they provide instant access to data stores that are pro-actively curated.

Off-line data sources may not be actively maintained, or may only release edits periodically (such as with a new publication) and typically include information contained within texts, files, or databases which may have been produced by a lab during the research process. It is most likely that each off-line source differs in its storage format or layout, especially in the case of textural publications, and thus must have a bespoke parser written for it. This process is very time consuming and so these sources are currently avoided.

### Data curation and post processing

Data is collected currently on an ad-hoc basis, however when a new model is to be trained a snapshot of the database is taken so that continual data collection may occur. These snapshots are completely disconnected from the original data source, thus any updates to the database are not reflected in any dataset used by the model training process.

Post processing is the first step in data curation, and involves processing values and schema structure to assist with preparing data for curation. As an example, the AFLOW schema is mostly flat, however the Materials Project schema is nested. The Hadoken software prepares nested data by moving it to a simple normalised schema ready for the curation and matching process.

Data curation is a process that involves the careful selection and combination of data sources. Data sources may have differing, non-identical schemas applied to them, which will affect the storage and representation of underlying data. During the process, attributes from disparate data sources are matched where possible. Decisions must also be made about the treatment of nullable attributes. For example, it may be possible to replace null values with a default initialisation value, such as 0 for a null integer or an empty string for a null string. These decisions are realised in code, and applied in the software and underlying database schema.

All data collected must be curated, and this process involved dividing the data into two streams: high-quality and low-quality. Data must attain a completeness factor of 100% in order to be useful, so efforts are made to achieve this.

The completeness factor $F_{C}$ is the ratio of features that contain non-null values $F_{NN}$ to the total number of features $F_{Tot}$ in the dataset:

33 $$\begin{aligned} F_{C} = F_{NN}/F_{Tot} \end{aligned}$$

where $F_{NN}$ defines the number of features with non-null values and $F_{Tot}$ defines the total number of features present.

Data is considered high-quality if its $F_{C} > 0.9$, with the additional constraint that any missing attributes can be retro-fitted by reading them from associated files or calculating them directly.

Data is considered low-quality if its $F_{C} \le 0.9$. Records that contain missing attributes cannot be used by model training as they may mislead the model. Low-quality data is stored, but shelved for use later, as it may be possible to reconstruct missing attributes via ML, or, the data may be updated when matched with a future high-quality data source.

### API access

We present a lightweight REST API for accessing the machine learning models built from this curated data. The API is built on current industry standards supporting both JSON and XML data exchange formats. The full API definition is located on the Hadoken Materials website here: https://hadokenmaterials.io/Home/Api. Registration is required to use the API (https://hadokenmaterials.io/Account/SignUp) and is fast (and free), however registration is not required to use the web UI interfaces provided for each model.

Whilst use of the website is free, any use of the website or API for research purposes, commercial or otherwise, are governed by terms defined in the citing document available on the website. More information is available here https://hadokenmaterials.io/Home/Citing.

Upon completion of registration, an API key in the form of an 128-bit GUID is allocated and API access is granted to the entire platform. This API key must be presented during each request.

By way of example, a typical API request for a band gap prediction for the compound $Ca_{2}Cu_{2}Ge_{4}O_{12}$ follows:

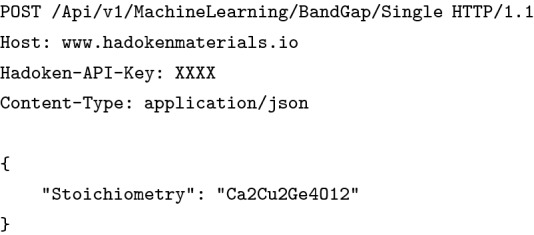



The response from this request (some headers omitted for brevity):

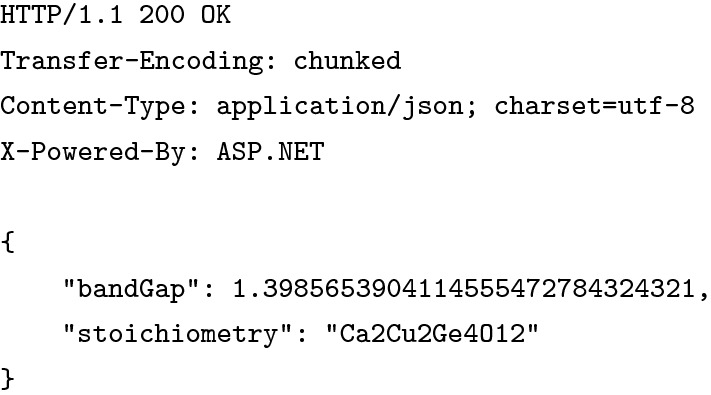



## API reference

Currently, the API supports a single version: 1. In the future, different versions will become available; to use those versions replace the current version number.

Table 15 details the entire API URI reference.

**Table 15:** Full URI reference-https://www.hadokenmaterials.io/Home/Api

Format	Method	Description
/Api/v{Version}/Species	GET	Retrieve a list of resources
/Api/v{Version}/Species/{Name}	GET	Retrieve a list of resources by name
/Api/v{Version}/Species?Start={Start}&Size={Size}	GET	Retrieve a list of resources constrained by arguments
/Api/v{Version}/Species/Species/{GUID}	GET	Retrieve a single resource by unique identifier
/Api/v{Version}/Compounds	GET	Retrieve a list of resources
/Api/v{Version}/Compounds/{Name}	GET	Retrieve a list of resources by stoichiometry (Fe2In1P1)
/Api/v{Version}/Compounds?Start={Start}&?Size={Size}	GET	Retrieve a list of resources constrained by arguments
/Api/v{Version}/Compounds/Compound/{GUID}	GET	Retrieve a single resource by unique identifier
/Api/v{Version}/Simulations	GET	Retrieve a list of resources
/Api/v{Version}/Simulations/{Name}	GET	Retrieve a list of resources by stoichiometry (Cu1In1Se2)
/Api/v{Version}/Simulations?Start={Start}&?Size={Size}	GET	Retrieve a list of resources constrained by arguments
/Api/v{Version}/Simulations/Simulation/{GUID}	GET	Retrieve a single resource by unique identifier
/Api/v{Version}/MachineLearning/BandGap/Single	POST	Compute a prediction from the posted data
/Api/v{Version}/MachineLearning/BandGap/SpaceGroupGeometry	POST	Compute a prediction from the posted data
/Api/v{Version}/MachineLearning/BandGap/SpaceGroupHighSymmetryDerived	POST	Compute a prediction from the posted data
/Api/v{Version}/MachineLearning/FermiEnergy/Geometry	POST	Compute a prediction from the posted data
/Api/v{Version}/MachineLearning/GapType/SpaceGroup	POST	Compute a prediction from the posted data

Table 16 details all optional query string parameters used by the API.

**Table 16:** Optional query string parameters

Parameter	Default	Minimum	Maximum
Size	100	1	200
Start	1	1	2147483647

### Machine learning API URI reference

#### Band gap-single feature

URL format: /api/vVersion/MachineLearning/BandGap/Single

JSON fragment template:

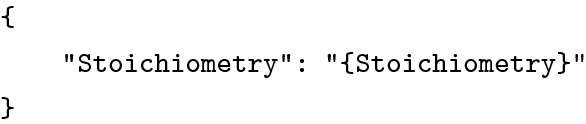



JSON fragment example:

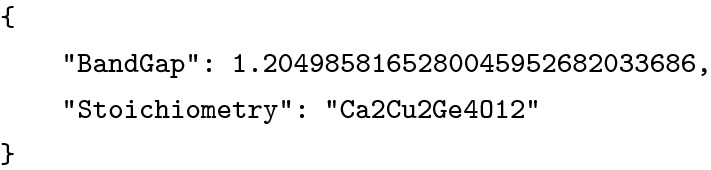



#### Band gap-minimal features

URL format: /api/vVersion/MachineLearning/BandGap/SpaceGroup Geometry

JSON fragment template:

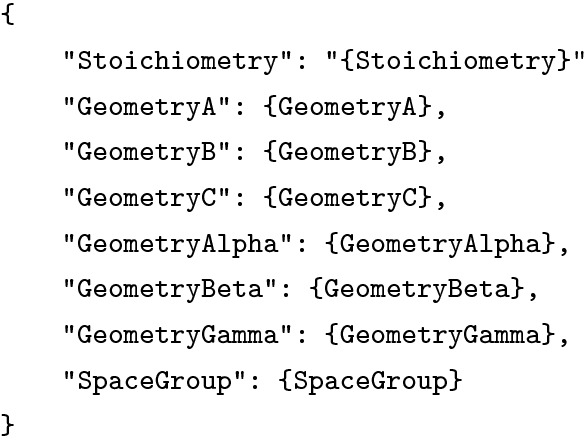



JSON fragment example:

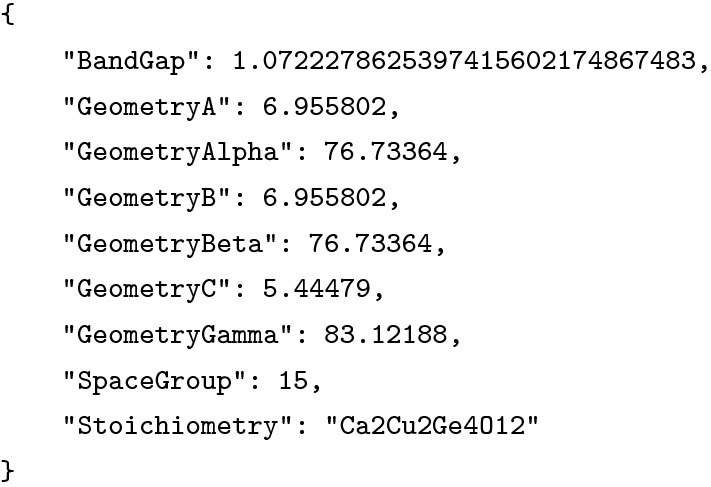



#### Band gap-maximal features

URL format: /api/vVersion/MachineLearning/BandGap/SpaceGroup HighSymmetryDerived

JSON fragment template:



JSON fragment example:

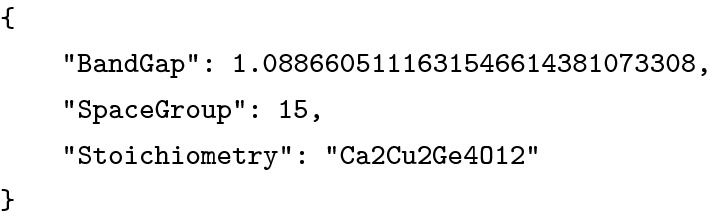



#### Fermi energy

URL format: /api/vVersion/MachineLearning/FermiEnergy/Geometry

JSON fragment template:

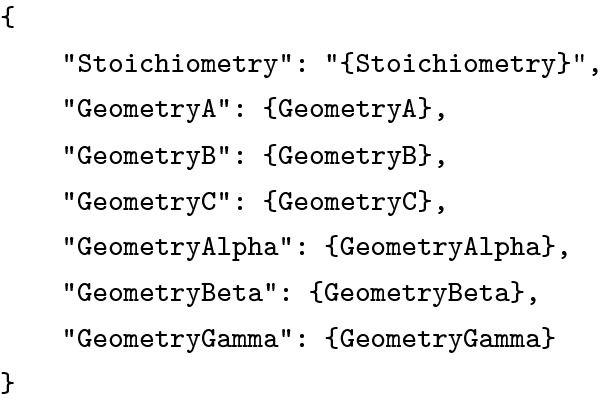



JSON fragment example:

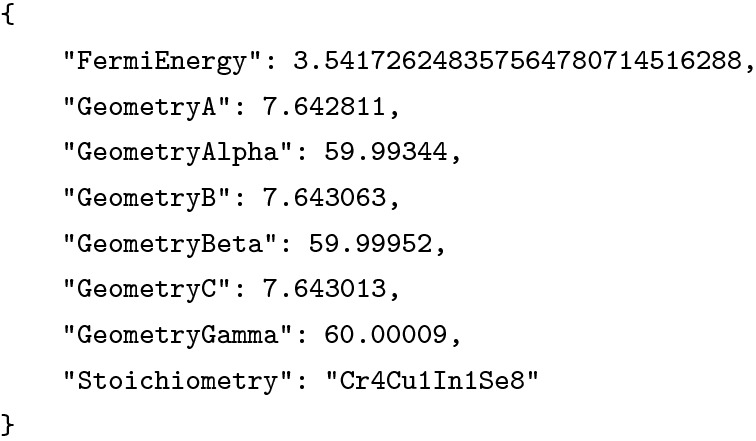



#### Gap type

URL format: /api/vVersion/MachineLearning/GapType/SpaceGroup

JSON fragment template:

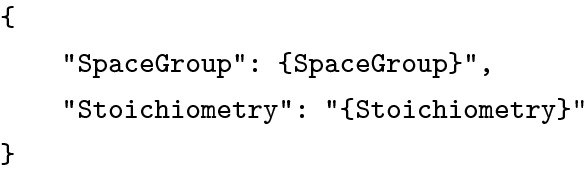



JSON fragment example:

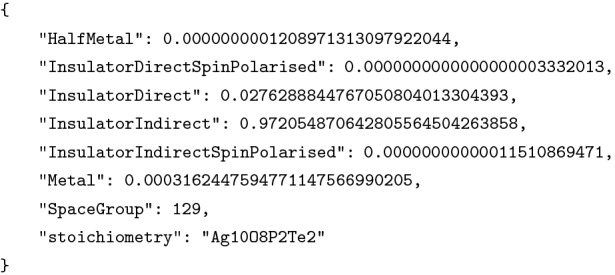



## Machine learning web URI reference

Visit the URLs listed below to use the corresponding ML model via a web UI.

### Band gap-single

Compute the $E_{g}$ from stoichiometry only. https://www.hadokenmaterials.io/MachineLearning/BandGapSingle

### Band gap-space group, geometry

Compute the $E_{g}$ from stoichiometry and geometry (cell lengths and angles). https://www.hadokenmaterials.io/MachineLearning/BandGapSpaceGroupGeometry

### Band gap-space group, derived

Compute the $E_{g}$ from stoichiometry and values derived from stoichiometry. Note for this model, only the stoichiometry is required for operation. https://www.hadokenmaterials.io/MachineLearning/BandGapSpaceGroupHighSymmetryDerived

### Fermi energy-geometry

Compute the $E_{F}$ from stoichiometry and geometry (cell lengths and angles). https://www.hadokenmaterials.io/MachineLearning/FermiEnergyGeometry

### Gap ttype-space group

Compute the gap type from stoichiometry and space group. https://www.hadokenmaterials.io/MachineLearning/GapTypeSpaceGroup

## Conclusions

In this paper we show that it is possible to develop a number of highly accurate ML models to inexpensively predict the properties of materials using information previously generated from computationally expensive simulations.

The ML models demonstrate that a stoichiometry definition alone is a high value feature, containing (in most cases) all the information required to accurately compute the band gap associated with that material. Initial experimenting has demonstrated that the addition of other features (such as Density or Total Atomic Weight) has yielded little, if any additional accuracy. This suggests that DFT computation may not be required to perform this type of calculation.

The prospect of fast, efficient DFT-free computation of materials properties using only consumer hardware is tantalising and implies that further investigation into properties implied by stoichiometry related to $E_{g}$ is required. This development could in turn greatly reduce the amount of time spent on simulations, managing simulation software, and budgets spent on supercomputing.

This project also lays the foundation for expansion to the prediction of other materials properties in the future using a similar process, and the development of an industry standard platform for the production development of said models should facilitate the exhaustive profiling of compounds to develop novel materials by the wider research community in general.

## Data Availability

Hadoken API: https://www.hadokenmaterials.io/. Additional resources: https://github.com/carlyman77/MaterialsDiscoveryML

## Abbreviations

ANN: Artificial Neural Network
API: Application Programming Interface
ARC: Australian Research Council
DFT: Density Functional Theory
DL: Deep Learning
HOMO: Highest Occupied Molecular Orbital
HT: High Throughput
JSON: JavaScript Object Notation
LUMO: Lowest Unoccupied Molecular Orbital
MAE: Mean Absolute Error
ML: Machine Learning
PBE: Perdew-Burke-Ernzerhof
RAD: Rapid Application Development
REST: Representational State Transfer
RMSE: Root Mean Squared Error
SQL: Structured Query Language
UI: User Interface
URL: Uniform Resource Locator
VASP: Vienna Ab initio Simulation Package
XML: Extensible Markup Language
